# Parents of Adolescents with Type 1 Diabetes - Their Views on Information and Communication Needs and Internet Use. A Qualitative Study

**DOI:** 10.1371/journal.pone.0062096

**Published:** 2013-04-23

**Authors:** Sam Nordfeldt, Teresia Ängarne-Lindberg, Maria Nordwall, Barbro Krevers

**Affiliations:** 1 Division of Child and Adolescent Psychiatry, Department of Clinical and Experimental Medicine, Linköping University, Linköping, Sweden; 2 Center for Medical Technology Assessment, Department of Medicine and Health Sciences, Linköping University, Linköping, Sweden; 3 Division of Paediatrics, Department of Clinical and Experimental Medicine, Linköping University, Linköping, Sweden; 4 Paediatric Clinic, Vrinnevi Hospital, Norrköping, Sweden; 5 Division of Physiotherapy, Department of Medical and Health Sciences, Linköping University, Linköping, Sweden; Tehran University of Medical Sciences, Islamic Republic of Iran

## Abstract

**Background:**

Little is known about parents’ views on the use of online resources for information, education and support regarding childhood type 1 diabetes (T1DM). Considering the rapidly evolving new communication practices, parents’ perspectives need to be explored. The main purpose of this paper was to explore parents’ perceptions of their information-seeking, Internet use, and social networking online. This applied to their everyday life, including the contexts of T1DM and contact with peers. A second aim was to identify implications for future development of Internet use in this respect.

**Methodology/Principal Findings:**

Twenty-seven parents of 24 young persons aged 10–17 with T1DM participated in eight focus group interviews during their regular visits to a county hospital. Focus group discussions were video/audio-taped, transcribed and analysed using inductive qualitative content analysis. Self-reported demographic and medical information was also collected. A main theme was *Finding things out*, including two sub-themes, *Trust* and *Suitability.* The latter were key factors affecting parents’ perceptions of online resources. Parents’ choice of information source was related to the situation, previous experiences and knowledge about sources and, most importantly, the level of trust in the source. A constantly present background theme was *Life situation*, including two sub-themes, *Roles and functions* and *Emotions and needs*. Parents’ information-seeking regarding T1DM varied greatly, and was closely associated with their life situation, the adolescents development phases and the disease trajectory.

**Conclusions/Significance:**

Health practitioners and system developers need to focus on creating trust and suitability for users’ needs. They should understand the children’s diverse needs, which depend on their life situation, on the children’s development, and on the disease trajectory. To enhance trust in online health information and support services, the participation of local practitioners is crucial.

## Introduction

Life with type 1 diabetes (T1DM) requires extensive self-care and comprehensive knowledge [1]. Parents of children with T1DM have to assume great responsibility for their child’s management of the disease, and medical as well as psychosocial factors affect their everyday life [2], [3]. Altogether this increases the risk of family conflict and parental distress, and even burnout symptoms [4], [5]. In striving for autonomy, adolescents with T1DM need distance from their parents, yet parents need to continue giving support [6]. Continuing positive and collaborative parent involvement during the transition into adolescence enhances glycaemic control and quality of life [3]. This implies that support for parents is needed concerning how to maintain a positive role in the relationship with their child [3]. Novel and effective approaches are required, such as different types of parent education and support regarding coping skills [7]. These approaches should be embedded in health care services in order to reduce parents’ stress and empower them in supporting their child’s self-care [7], [8].

According to adolescents’ and parents’ perspectives on quality of care, improvements are needed regarding information and access to services [9]. In a multinational survey, receiving information at diagnosis and having access to multiple sources of information later on were associated with better outcomes from the perspectives of young T1DM adults and parents of children and adolescents with TIDM [10]. The most frequently used information sources were diabetes medical teams, websites and diabetes associations, with the diabetes team being the main source. In addition, as many as 75% of parents felt that being able to talk to peer parents was important or fairly important.

Web-based services have been suggested as one way to meet the parents’ diverse needs for knowledge and support [11]. The Internet is a rapidly emerging source of information, health services, and peer support through online networks and forums [12], [13], [14]. Numerous online websites and parental discussion groups exist related to childhood T1DM [15], [16], [17]. The umbrella term ‘Web 2.0’ describes a range of widely used Internet applications to enhance participation, collaboration, openness, social networking and sharing information with peers [18]. Such new technologies have great potential to enhance health information delivery and exchange when needed, and this includes use of new mobile devices [16], [17], [18]. Hence, much remains to be learned for practitioners and system developers [19], [20]. To ensure successful implementation of web-based services in healthcare, the perspectives of parents need more attention.

The major aim of this study was to explore parents’ views on their information-seeking, Internet use, and social networking online. This applied to their everyday life, including the context of T1DM and contact with peers. A second aim was to identify implications for the future development of Internet use in this respect.

## Methods

### Ethics Statement

This study was performed in accordance with The Declaration of Helsinki and The Swedish Act concerning the Ethical Review of Research Involving Humans (SFS 2003∶460). The parents received an information letter about the study from the researchers. Verbal consent was given to a nurse, recorded by her in written notes and reported to the researchers before each group interview. The parents were informed in the letter about confidentiality and their right to withdraw without giving a reason. This was repeated by the researchers in personalized conversations with eligible participants, thus confirming their consent prior to them entering the interview room. The researchers who collected data (SN, BK) and performed the analysis (SN, TÄL) had no other relation to the participants or their care process. Furthermore all data were sampled and analysed anonymously; no consent forms were used for participants. Each participant was compensated with a cinema ticket. This study protocol was approved by the Regional Ethical Review Board of Linköping, Permit Number 2011/167-31. The Ethical Review Board did specifically approve the consent procedure.

### Sample and Setting

A consecutive sample of fathers and mothers of adolescents with type 1 diabetes (T1DM) visiting their County hospital in south-east Sweden for a scheduled medical check-up were eligible. A strategic sampling of parents was made based on the age and gender of their adolescents. In all, there were eight groups of parents, split according to the adolescents’ gender and ages 10–11; 12–13; 14–15; 16–17 years respectively. A total of 17 women and 10 men participated, mean age 43 years, range 30–56 years. The participants were parents of 11 girls and 13 boys aged 10–17 years, taking 2–7 insulin doses and having 0–6 blood glucose checks daily, recent HbA1c range 38–91 mmol/mol (DCCT 5.6–10.5%) and T1DM duration 0.5–11 years. Additional self-reported characteristics are presented in table 1.

**Table 1:** Characteristics of the participants as self-reported on a data sheet (N = 27).

Characteristic		n
**Highest education level**	Compulsory school	2
	Secondary or vocational school	19
	University education	6
**Sources used for support/information** **related to T1DM**	Diabetes nurse	24
	Internet - unspecified	14
	Diabetes sites	5
	Diabetes organizations	3
	Friends	3
	Family	1
**Internet access**	At home	27
	At work	15
**Internet use**	Daily	22
	Weekly	4

### Procedure

Considering the exploratory aim in a rather new field the study had a qualitative inductive and descriptive design. Focus group discussions were used, allowing the researchers to examine participants’ points of view as they shared their experiences. Obtaining data from eight discussion groups provided rich and broad information that helped the researchers clarify and understand complex phenomena [21], [22].

The interviews were held in a physically familiar clinical care context but with no clinical staff present. They lasted for 60–90 minutes, and were conducted by two of the authors alternately (SN, BK). The interviews were recorded and everything was transcribed verbatim, including markers for factors such as notable body language and change of voice. An interview guide was used to ensure that the same basic lines of inquiry were pursued with each group interviewed [23]. Guiding questions focusing on information-seeking behaviour, Internet use, and use of social media in general, and particularly regarding T1DM, were asked. Questions also focused on the parents’ experiences and need for contact with peer families. Examples of guiding questions are found in table 2. A web portal targeting young people with T1DM and their next of kin was demonstrated at the end of the discussion.

**Table 2:** Examples of focus group guiding questions from the interview guide.

Area	Sample question
**Seeking information**	How do you find things out? How do you gather information?
	How do you decide what (sources) you can trust?
	What are your experiences of Internet use in general?
	What are your experiences of Internet use for contact with others?
	What are your experiences of Internet use for contact with healthcare?
	What’s important for quality? What’s important for trust?
**Peer contacts**	What are your experiences of contacts with other parents of children with diabetes?
	What do peer contacts mean to you? What’s important to you?
**T1DM sites**	What are your experiences of diabetes websites?
	What could make you use, or not use, such a site?
	What role, if any, could communication over the Internet play for you as a parent of a child with diabetes?

### Data Analysis

Based on the exploratory aim of the study, an inductive qualitative content analysis was performed. This method can be used to examine patterns and themes in order to elucidate the content of transcribed interviews, texts, narratives, letters, documents, protocols and media [24]. It allows the study of both apparent and latent content, which, in turn, allows deeper insights and the tracking of emerging or new perceptions.

Initially the analysis was performed by two of the authors (SN, TÄL) independently. The videos were viewed and the transcripts were read several times by each of them so they could achieve familiarity with the contents. Statements with similarities were clustered and summarized into tentative themes based on their emerging contents. Relevant phrases recognized as ‘meaning units’ were labelled with codes. Codes with similar or identical meaning were merged into sub-themes, and sub-themes with related meaning were clustered into larger themes. The tentative sub-themes and themes with all their respective statements were reviewed in detail. Unclear statements were further explored by again reading them in the full original context.

In a second phase, to ensure reliability, SN and TÄL conducted open comparisons. Before the sessions they both read all the primary data and the material emerging in the analysis. Any discrepancies were resolved through discussion. Through iterative in-depth discussions, stepwise re-categorizations and repeated validations vs. the complete primary data, a logical and complete structure eventually emerged. Thus all sub-themes and themes were validated through repeated systematic reviews of the material.

In a third phase, the above preliminary analysis was presented and iteratively discussed with all authors. In this later and final stage of validation, the complete sequences from the original material were again reviewed in their original context and condensed into final themes, and some final adjustments were made.

Both apparent and latent content were considered important [24]. Throughout all phases, several quotations to confirm and illustrate each theme and sub-theme were considered in order to facilitate validation by all authors. Finally a limited number of quotations to illustrate and confirm the themes and sub-themes were selected.

BK was experienced in the methods used for data collection and analysis as well as in caring and care research. MN was experienced in clinical research and the care of the target group. TÄL was experienced in the methods used for data collection and analysis. SN was experienced in the methods used for data analysis, as well as in clinical research and the care of the target group. The risk of bias due to the authors’ preconceptions or potential expectations was prevented as far as possible through the repeated validations against the primary data and the in-depth supervision sessions in the author group.

## Results

**Figure 1:**
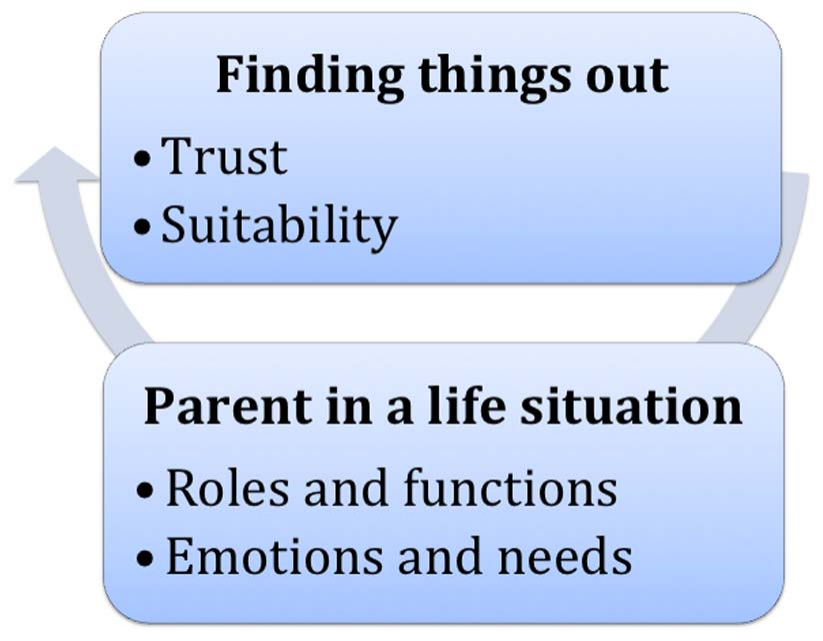
Overview of themes and sub-themes.

An overview of themes emerging from the data is found in figure 1. A major theme was *Finding things out*, with the two sub-themes *Trust* and *Suitability*. Parents described a wide range of ways to find things out. Their choice of source was related to the situation, previous experiences and knowledge about sources and, most importantly, the level of trust in the source. A constantly present background theme was *Parent in a life situation,* with the sub-themes *Roles and functions* and *Emotions and needs*. Parents’ needs for communication and information regarding diabetes-related issues varied greatly, being closely related to life situation. They were also dependent on the adolescents development and the disease trajectory.

### 1. Background Theme: Parent in a Life Situation

A background theme of diversity and complexity appears over a long time span in a life situation, including different phases of life with the disease. Each parent is involved with unique experiences, challenges and struggles. Thus parents are living in their relational systems including various roles and functions. Parenting their child with diabetes, guiding the child through varying medical scenarios as well as different phases of childhood and adolescence, together represent only one part of their life situation. The different functions and roles in which parents array themselves, and shoulder the responsibility for, are parts of a life situation that can be characterised as having two sides, different but inseparable like the two sides of a coin. One side expresses the actual situation in life and the parent’s identity, with functions and roles to deal with, and the other expresses emotions and needs in relation to these roles and functions.

#### 1.1 Roles and functions

Parents expressed themselves as being in different roles, implying they faced a range of different situations. There were descriptions of situations of being a mother or a father of not only a teenager, but also a teenager with diabetes. There were further descriptions of all the difficulties the parent had to deal with in the collision of roles they had to adopt when dealing with the teenager. The parent was a partner, someone’s friend, a person with other interests, a parent of other children, and the parent of a disabled teenager with diabetes.

*-For us this is more the identity of being disabled that’s come to the forefront and it’s important for (name of child) to take part, and thus she was in a regular class before, and then she changed in (a later grade), so it’s her identity, more about being disabled, not being normal you could say, so she struggles a lot with that. So for us her diabetes is like just on top of everything else (Mother)*

The challenges facing the parents went through phases of the disease trajectory and their children’s development. Sometimes the challenges were greater than at other times, also in relation to the parents’ life situation and how those around them responded. This implies varying needs for information and support over time.

*-But circumstances change for you, and maybe you enter a new phase, like now when we are on the way in, the girls are starting to enter puberty and a lot starts to happen and the blood sugar is flowing and I don’t know why. Then this need to get information is reborn and then maybe you go out and try to get information… (Mother)*

#### 1.2 Emotions and needs

Emotional challenges arose in relation to the demands of life situations with diabetes. Initially, when there was much new information to take in, there was emotional shock, and this was followed by a phase of sorrow and struggle in order to get back to an ordinary life.

*-In the beginning there is much, you get very much, and then it is quiet somehow (Mother 3)*

*-In the beginning there is much, then it is quiet? (Researcher)*

*-…yes well when you were admitted to the hospital, there was a lot of information you got and hand-outs and all this, and then you came to visit the daytime ward and then you had to manage by yourself pretty soon, rather quickly… (Mother 3)*

*-Pretty soon all by yourself (Mother 4; mothers 1–3 nodding)*

*-At first you didn’t realize how much there was, it involved everything but it… took a while before we… first there was a kind of shock or sorrow, then what should we do to get things to work as well as possible to get back to the life the child had before? We’re struggling for it to be that way… (Mother 4)*

There were sources of worry that persisted over longer periods of time. Some parents felt as if no matter what they did, after having tried everything, their child’s blood glucose still fluctuated and they had to live with continuous worry about the consequences. Others felt as if their adolescent had not accepted the diabetes, even after a long duration of the disease, thus increasing their worries. Parents also said that just like other children, their children sometimes forgot; for instance, they ate the wrong things, or forgot doses. As parents, they could not glue themselves to their child and control everything. In this context the regular check-ups at the clinic were perceived by some as a sort of “police-control,” with an outcome of approval or non-approval.

*-…Just what you were talking about that you feel that all the, what should I say, responsibility and also a little blame falls on us, and then you think okay, we’re going to be more alert, but I have in fact talked with him. Of course you give them information at home and now they know what’s important, it’s really important, but how in the world can it be your fault that they still don’t do it; they aren’t small children any longer… (Mother)*

Emotions emerged from these situations; feelings of worry mixed with frustration, dejection, a guilty conscience and fatigue, feelings sometimes perceived as not being permissible.

*-… but you can’t say to your child “goddammit I’m so tired of keeping track” I mean…it’s even harder for the child, but I think, anyway like for me, just to be able to say “goddammit” (laughs). I mean you should be supportive of the child and then you can’t say “Damn, diabetes is such a pain” (Mother)*

Some parents found comfort in sharing these experiences and emotions with peers in the same situation. Others, however, toned down that need and said that it differed from time to time.

Parents also talked about the importance of setting limits for parent involvement, pointing out the necessity of leaving things to the child in the long run. They reflected, in this context, on the fact that practitioners often kept talking to the parent, and that their adolescent did not respond. This left everything to the parent during the hospital visit, and thus parents continued bearing the responsibility. Parents suggested that practitioners talk directly to the adolescent without the parent being present, gradually reducing the involvement of the parent.

*-Everything falls on us… (Mother 4)*

*-Yes there’s a great deal of focus on what I do wrong or what I can do. It’s better that now Mom can go out because maybe he would talk to the doctor himself if I was not there. Maybe he thinks he’ll be punished by Mom if he says ‘I went and took that fruit or sandwich’. It would be nicer if they listened but okay, now Mom is leaving so we can discuss things and see if we can come to any conclusions, I think that would be really good (Mother 1)*

Another issue that was emphasized was the need to keep some things in life separate from diabetes in order to maintain a balance between a normal life and a special life due to the demands of the disease, aiming at a life as close as possible to that of everyone else.

*-Yes because you have to cope with it in some way or other your whole life I think there also have to be days where things are normal. Even though for them all the injections are like normal (Mother 1)*

*-I understand what you mean (Mother 2)*

*-And like it’s their ordinary daily life and mine too as long as (her child) is living at home with me, that’s how it is, this is how our daily life is, but then sometimes it’s like…well to just be and not nag about that blood sugar. What was it? Was it good or…? (Mother 1)*

*-Yes, life must also be able to be about something else (Mother 2)*

*-Yes (Mother 1)*

*-Uh-huh (Mother 2)*

### 2. Major Theme: Finding Things Out

Parents described a wide range of ways to communicate, retrieve information – to find things out. Different channels were used for different things; physical meetings, telephone meetings, books, brochures, and the Internet. The choice of source was related to the present situation, the seriousness of the present situation, previous experiences and knowledge about sources and, in particular, the level of trust in the source. Thus the major theme, *Finding things out,* was based on the two sub-themes, *Trust* and *Suitability,* as follows.

#### 2.1 Trust

Parents appreciated having access to reliable facts and information, receiving positive hints and advice, and identifying with the experiences of others. For safe information and guidance, practitioners at the clinic were the parents’ major source. Parents talked about supportive, trustful long-term relations with their diabetes team. They could rely on the hospital in a crisis, for support and advice, and even for getting their child admitted when necessary. Trust in the practitioners as sources of information was high.

*-I think it’s worked well. You often have to call in an emergency situation, since things can happen on weekends and then you’re a little… and I think there’s always been someone who can help or someone who can answer questions and those who are on call… (Mother)*

The need to share things with peers varied from person to person, and as mentioned earlier, this might change from time to time. As in other relations, trust appeared to be important. When meeting other parents in groups at the clinic, they emphasized the importance of a permissive, trustful atmosphere.

*-.that there’s a permissive atmosphere, that you dare to say that this is darned hard or that you just don’t have to, that you just don’t feel the need to have everything good the whole time, that there’s this forum where you actually also can say that this is really hard (Mother 1)*

*-When you talk with others it’s often the case that they all say that it’s going really well and they are so good and so you feel that yes but it doesn’t always work so well (Mother 2)*

When it came to online information, in general the level of trust was low; parents emphasized the need for a critical attitude. They talked about different strategies, about how to evaluate online information, comparing similar content from different sources and individuals. Close examination of the source was important, i.e. “About” and “Contact information” on the site, examining the update level, and in forums checking the status of the individuals and the number of participants involved in a discussion. In the end, it was a matter of trusting their own judgment.

*-I usually look at “search”, “contact us”, I usually look at those and see if… (Father)*

*-Like where are they (Mother 2)*

*-Well if it seems to be serious or how it is, so it’s fairly often when you look for something special or find some new company or something, well I think so and then you can Google it and the name if you want (Father)*

*-What I think is good, you could say, in many respects is that you hopefully also find information and facts that are hopefully rather new, I mean fairly recently updated if these are serious pages, thus it’s also a big advantage that it’s not some old literature or something old, it isn’t literature that’s not updated, so you can be fairly sure that if you find a good site that the latest can… (Mother 2)*

*-…have to check a little about when it’s written and the like because sometimes you can also find old things that come up first so that yes… (Father)*

For trust in online health-related information, County Council (local government and healthcare provider) websites were preferred because the parents knew the information was based on facts and that health professionals stood behind this information and/or appeared in person on the website. Also, municipal (smaller local governments) websites and the national pharmacy website were mentioned as examples of safe sources. Parents said they could trust someone online, such as a practitioner, after previously having met that person. Knowing who was saying what enhanced trust.

#### 2.2 Suitability

When trying to find out about something, parents used methods they were used to, comfortable with, and felt were appropriate for the purpose at hand. Parents “Googled,” at least to begin with. Then the choice of source depended on the situation, the subject and its seriousness, previous experiences, knowledge, and the level of trust. Thus, the meaning of suitability varied for different parents.

For individual diabetes-related issues, parents often preferred a personal meeting or telephone contact pointing out that communication was a broad concept that also comprised body language, eye contact and closeness, the atmosphere in the room, etc. They said that a person’s long experience was difficult to communicate only through books, brochures or the Internet. Parents regarded the use of online text messages as inferior to telephone contact due to the risk of misunderstandings, difficulties in writing questions in the right way, and the need to wait for an answer, and it was felt that direct verbal communication was more appropriate, i.e. a better fit, regarding complicated issues and nuances. However, appropriate ways of using the Internet were said to simplify and enhance communication, offering new and easily accessible information on areas of interest, but also access to others’ perspectives, and other cultures. Some of the parents had had no such positive experiences, but on the whole a wide range of beneficial examples from use of online communication were mentioned. They gave some ideas for using complementary electronic communication to enhance information and support when needed.

*-On the other hand I want closer contact with someone who can provide information and help with support, because I think that’s a bad part of healthcare. There’s like a telephone number and then you’re transferred onward and then you push one button after the other and then you’re called the next day instead… (Father 2)*

*-…If you could meet some way over the net, I don’t know how that would work, if it’s regular email or if it’s like Skype or whatever it is, that ought to make it easier in some way for everyone (Father 2)*

*-I’d like there to be more doctors, more nurses, more money for healthcare and that I could come here and see the doctors instead of looking at computers (Mother 1)*

*-The one doesn’t have to exclude the other (Father 1)*

*-…that I don’t know, computers are still technology and it’s not the same as talking with a person, it’s as impersonal as anything can be (Mother 1)*

*-No, I’m no computer-literate person either, I like meeting people better but (Mother 2)*

*-Uh-huh, exactly (Mother 1)*

*-But maybe sometimes you can, maybe many visits are unnecessary? (Mother 2)*

For example, since individual users can meet with family and friends, even internationally, via free videophone (Skype, for example), they requested to have this option for communicating with their practitioners.

*-I talk with people on Skype, with many (Mother)*

*//*

*-How would you like to be able to use Skype with C [a nurse in the diabetes team]? (Researcher)*

*-Perfect, and my God I would like that, because it’s like sitting across from one another and talking. I don’t need to write, you don’t have to, you talk with each other, that’s the best there is (Mother 1)*

*-But you also do that on the telephone, right? (Father)*

*-Yes okay, the telephone is the same thing, but here you get contact right away (Mother 1)*

*-You get a little closer (Father)*

*-You get closer (Mother 1)*

*-…a higher level you could say (Father)*

*-Uh-huh, much better, oh absolutely, I’d like that (Mother 1)*

In contrast to the expressed need for personal contact, electronic communication offered, in some respects, an easier way to say things. Some parents felt they could more freely express themselves in writing, and were also able to think more about the issue before asking questions. Parents also expressed an interest in what others had asked and the answers they had received.

*-I think it’s complicated to write a question and then wait for the answer, I like to call or I search further. I can also imagine maybe logging on and listening to or reading others’ questions and answers, then I’d get more out of it than if I first have to formulate the question and then wait for the answer… (Mother)*

Finding things out through sharing experiences with peers was comforting for some parents, making them feel less alone, enabling them to talk about things and feelings as they were. A currently widespread social network online service (Facebook) was used to varying degrees, from daily to weekly by parents, but in different ways; for example, for staying connected across geographical borders, for enhancing communication and saving time, and for some even as an open diary mirroring their life. Parents rarely visited a specific forum – at the time of interview, Facebook was their forum, where parents and children connected with peers who would otherwise not have been communicating.

*-So you have contacts, do you get new contacts? (Researcher)*

*-Uh-huh (Mother 4)*

*-Yes (Mother 1)*

*-Uh-huh (Mother 3)*

*-Yes (Mother 2)*

*-And then when I’ve used it, you can enter old contacts, where people search for you and so on through the forum but… yes… (Mother 4)*

*-It’s the same, there are also so many different groups to be in, whatever it involves, whether it’s diseases or things to do, there are different groups. Maybe it’s people with the same feelings as you and can write something and maybe get a little response, and the same thing if someone else writes something that you know something about, you can respond and also help others, so it’s very broad and there’s a great deal of variation. It’s not just talking with friends but also finding information there or the like. You can ask others who’ve been in the same situation with things and get other perspectives on things (Mother 1; Mother 4 says uh-huh now and then)*

Blogs were visited by parents to pick up ideas and hints, but also to follow and to identify with known or unknown persons in special life situations such as divorce or critical phases of life with severe illness. A blog could be used for sharing things that were difficult to say in personal contacts. Experiences of online forums varied. Some parents found them supportive, providing helpful information, while others perceived them as complicated, messy or giving too much contradictory information. Certain forums were perceived as more serious, where someone was building up and caring for the contents in an educational manner. A forum was considered helpful if there was a high level of activity, and the parents expressed positive thoughts about forums on diabetes with doctors. A high activity level and a supportive way of communicating were also requested on the part of practitioners.

*-No, it’s uninteresting if you write something and three months go by or if you see that the last person who wrote something did so two months ago (Mother 2)*

*-Yes, exactly (Mother 1)*

*-Who should be active, should it be parents or should it be the young people, should it be healthcare professionals, who should be active? (Researcher 1)*

*-Well, both parents and young people and children (Mother 1)*

*-Should health professionals have some kind of hands off? (Researcher 2)*

*-No, it would be really good if they were also active (Mother 1)*

*-[… ] It also depends a little on how they contribute, if they go in and correct everything then there isn’t… there’s no point, then you also end up that you don’t want to expose yourself because then you’ll get told hello, like to find routines or something… (Mother 2)*

Supporting someone else could be rewarding, and individuals even networked internationally across cultural borders. Some possible prerequisites for engaging in a forum were mentioned; a specific need for somewhat general information, a high level of seriousness on the site, a personal sense of having something from one’s own experience to share for the benefit of others – and perceiving use of the forum as smooth and easy, and not too time consuming.

*-…I think it’s usually like rings on water, that I search for something and get an answer and then there may be someone else who answers something else, then I’m tempted to want to help the person of course, based on my experiences… (Mother)*

## Discussion

This study highlights parents’ different and varying needs for information when living with a child or adolescent with T1DM. Parents’ information-seeking relates to their life situation in general, but also to their child’s development and the different phases of the disease trajectory. Personal contact with practitioners is of central importance to parents, due in part to the complexity and uniqueness of situations involving T1DM. Overall, the results confirm the role of electronic communication as complementary and important [25].

Understanding the diversity and variability of this target group is important in developing information and communication technology (ICT) resources for the group. Trust and suitability for users appear as key elements in a relational interplay where understanding the background life situation, roles and emotions is crucial. The exchange of information between parents and the T1DM team practitioners often has a high level of perceived suitability and trust. Thus, practitioner participation is important for enhancing trust when information is obtained electronically e.g. from websites and discussion boards [15], [16], [20].

### Parent in a Life Situation

Parenting a child or adolescent with T1DM involves the same hardships and reasons for rejoicing as parenting other children and adolescents. However, in addition, there is the disease, which complicates things from time to time with varying intensity and affects all members of the family [26], [27], [28], [29], [30].

The parents’ need for, time for, interest in, and openness to information related to T1DM differ and also change over time. These factors are affected by circumstances such as earlier experiences, the phase of the disease trajectory, the child’s age, additional diseases, a changed family situation, worries and feelings of insufficiency, parental work, and other children in the family. In addition, parents wish for a life that is not always related to T1DM.

Thus, in order to understand parents’ needs and information-seeking behaviour in general, and concerning T1DM in particular, we have to take an interest in their whole life situation. This means life and all it implies - combined with the struggle for optimal care of their children’s’ or adolescent’s T1DM and a healthy way of living.

### Trust

Based on earlier experiences and personal judgments, different strategies are used to assess the trustworthiness of online sources. According to previous studies, strategies include comparing sources, checking the sources out, and checking facts when meeting a known practitioner [31], [32], [33]. Some have concerns about who, and what interests are behind a website; hence, sites where practitioners are involved and sites maintained by government healthcare providers (County Councils in Sweden) are mentioned as trustworthy sources.

Face-to-face contact with a health professional has previously been considered to be twice as important a channel for health information as the Internet [25]. When trying to find things out, trust in the source is important for parents, and even more so in the context of their child’s or adolescent’s’ T1DM. The practitioners constitute the most trusted source, while online sources in general appear to be trusted to a much lesser extent. Trust in practitioners refers to interpersonal trust in a particular practitioner, and/or social trust in the healthcare system or the medical profession [34]. Patient-physician communication is a considerable part of clinical practice [35]. Thus, trustworthiness in electronic communication can be derived if practitioners are involved in the modality used [20].

### Suitability

As previously reported, people find both advantages and disadvantages with electronic communication [31], [36]. To a great extent the parents’ choice of communication tools is based on a personal evaluation of previous experience and what fits with the situation. In sensitive and complex situations, personal communication is preferred, either through a physical meeting, by phone or, for some, by electronic communication. Parents emphasize the importance of personal meetings and the possible risk of misunderstandings with electronic communication. They also mention situations in clinical encounters where live communication may be hampered, implying a potential for complementary ICT options. Parents mention the possibility of more freely expressing oneself in writing, and being able to think more about the particular issue before asking questions.

Thus parents’ varying information and support needs require suitability in aspects such as timing, problem area, relation, trust and modality of communication. Whereas a T1DM portal with online facts appears useful to some, others may not perceive a need for it because they already feel well informed and confident in the relationship with their practitioners. Peers may play a role in support and mediation of information, but parents’ interest in peer contact varies greatly. Regarding emotional needs and the need for practical advice, social networking online is perceived as useful by some, including the option of helping others. However, others fear getting less helpful or even unpleasant responses.

Parents also feel that practitioners’ attitudes and communication style online need to be supportive and non-judgmental. This implies a need for practitioners to be aware of the mutual intergrading process in dialogues with patients, offline as well as online. Based on observations of nurses and children, intergradation has been described as how the phrases used in the information exchange merge gradually with one another, maintaining the integrity of all, through dialogues that constitute unbroken sequences of information exchange [37].

### Methodological Considerations

To enhance participation, we performed the interviews in a hospital setting familiar to the participants. The parents were informed of the researchers’ independence from the clinic before giving their consent to participate, and no clinical staff were present during the interviews. Prior to data collection the participants had not taken part in projects for implementing electronic communication regarding T1DM treatment. In accord with traditional care practice at the time, it might be that many parents simply were not used to thinking about ICT in the context of T1DM.

### Future Implications

Parents in this study see advantages and disadvantages in all means of information retrieval. There are also parents who never use the Internet at home, or describe being strongly selective when using it. To many, obstacles hindering use of ICT for T1DM-related issues are their personal habits and the fact that existing services are perceived as not good enough, along with a lack of trust in or positive experiences of ICT. High levels of trust and suitability are required for successful implementation of electronic communication, and even so, technical ICT solutions cannot be expected to meet real-world care and support needs completely. As previously suggested, Internet-based support and local support in the real world ought to complement each other [19], [38], [39], [40]. Indeed, human interaction cannot be replaced by technological solutions; instead the challenge is to develop technologies that enhance the qualities of human interaction. ICT offers possibilities and increased independence through freedom of choice, freedom of expression and flexibility in time and place.

Future resources should offer a flexible choice of ICT services. To start with, this could mean applying a wider range of already existing, widely used and simple technologies. Contact with practitioners when needed is of central importance to parents. For some, simply adding, for example, videophone, e-mail or chat room options would facilitate their communication. This study suggests that the general availability of such improved services for communication with healthcare practitioners may be of substantial benefit to many. Secondly, open interactive thematic information resources including facts and open discussion with practitioners may be of benefit to many [15], [16]. A key issue for successful design is to maintain trustworthiness [33], again implying the need for practitioners, website editors and parents to collaborate [15], [16], [20], [38], [40].

### Conclusions

The need for, and interest in information differs over time and is affected by parents’ whole life situation, including a life situation that is not always related to T1DM. Health practitioners and system developers need to focus on creating trust and suitability for users. To enhance parents’ trust in online health information and services, the increased participation of local practitioners seems crucial.
